# Complete Genome Sequence of Brevibacterium frigoritolerans Ant232, Isolated from Antarctic Snow

**DOI:** 10.1128/mra.00152-22

**Published:** 2022-04-07

**Authors:** Yong-Hoe Choe, Jong Ik Lee, Mincheol Kim

**Affiliations:** a Division of Life Science, Korea Polar Research Institute, Incheon, Republic of Korea; b Division of Earth Science, Korea Polar Research Institute, Incheon, Republic of Korea; University of Delaware

## Abstract

We report the complete and circularized genome sequences of Brevibacterium frigoritolerans Ant232, generated using a combination of Illumina and PacBio platforms. The high-quality complete genome consists of a circular 5,586,945-bp chromosome and a 305,498-bp plasmid, with G+C contents of 40.66% and 36.8%, respectively.

## ANNOUNCEMENT

Brevibacterium frigoritolerans was first isolated from arid soils of Morocco in 1967 and assigned to the genus *Brevibacterium*, which was established by Breed in 1953 (1). Many species of *Brevibacterium* are ubiquitously found in various environments, such as food, humans, saline soil, sediments, and marine environments (2–6). Several *Brevibacterium* strains have the potential for bioremediation of phorate in agricultural soils (7). Some *Brevibacterium* strains can tolerate abiotic stress, such as drought and salinity (8). However, only one complete genome sequence, that of B. frigoritolerans ZB201705, is available (8), which encouraged us to isolate and sequence the genome of B. frigoritolerans Ant232 to better understand the genetic properties of this species.

In this study, a bacterium named B. frigoritolerans Ant232 was isolated from a surface snow sample collected from inland Antarctica (74°48.399′S, 159°15.461′E) in November 2019. The snow meltwater was plated on 100% strength solid tryptone soy agar and incubated at 15°C for 3 weeks. Single colonies were selected based on morphology and/or pigmentation and were restreaked several times until pure cultures were obtained. The genomic DNA was extracted by harvesting cells on a plate cultured at 15°C for 5 days, using the MagAttract HMW DNA kit (Qiagen, Netherlands). After shearing of the genomic DNA to 20 kb, a long-read sequencing library was prepared using the SMRTbell template preparation kit v1.0 (PN 100-259-100; Pacific Biosciences [PacBio], Menlo Park, CA) and then sequenced in single-molecule real-time (SMRT) cells with PacBio RS II SMRT technology. A paired-end (2 × 150-bp) sequencing library was prepared using the Illumina TruSeq Nano DNA kit and then sequenced on the NovaSeq 6000 platform (Illumina, USA). The 664,123 total reads (read *N*_50_, 10,132 bp) produced by the PacBio RS II platform were assembled, whereas the Illumina platform produced 41,636,453 total reads. *De novo* assembly was performed by Flye v2.8.3 (9). The circularity of the genome and plasmid was confirmed by Circlator v1.5.5 (10). The generated assembly was quality assessed using BUSCO v4.1.2 (11), and the prediction of protein-coding sequences (CDSs) was performed using Prokka v1.14.5 (12). The functional annotation was conducted by DIAMOND v0.9.30 (13) with Gene Ontology using Blast2GO v4.1.9 (14). All tools were run with default parameters unless otherwise specified. The genome sequence of B. frigoritolerans Ant232 comprises a single 5,586,945-bp circular chromosome with a G+C content of 40.66% (814-fold coverage) and a single 305,498-bp circular plasmid with a G+C content of 36.8% (846-fold coverage). Annotation revealed 5,711 coding DNA sequences and 124 RNA genes (39 copies of rRNAs and 85 tRNAs). Analysis of pairwise similarities between full-length 16S rRNA gene sequences revealed that Ant232 has 99.73% sequence similarity to B. frigoritolerans DSM 8801. Information on the sequenced B. frigoritolerans Ant232 is summarized in Table 1. Average nucleotide identity (ANI) was calculated using the ANI calculator (https://www.ezbiocloud.net/tools/ani), which indicated that Ant232 was closely related to B. frigoritolerans ZB201705 (GenBank accession number GCA_004006475.1) (97.26%), DSM 8801 (GenBank accession number LUUS00000000) (97.01%), and FJAT-20673 (GenBank accession number LWJH00000000) (96.94%). The genome sequence of B. frigoritolerans Ant232 may provide a valuable resource for better understanding the mechanisms of adaptation to challenging environments.

**TABLE 1:** Genome characteristics of B. frigoritolerans Ant232

Feature	Data for:	
	Chromosome	Plasmid
Genome size (bp)	5,586,945	305,498
Genome coverage (×)	814	846
G+C content (%)	40.66	36.8
No. of CDSs	5,389	322
No. of rRNAs	39	0
No. of tRNAs	85	0
Circular^*a*^	T	T
GenBank accession no.	CP084539	CP084540

a Circular sequence: T, true; F, false.

### Data availability.

The complete genome sequence of B. frigoritolerans Ant232 has been deposited in GenBank under the accession numbers CP084539 (genome) and CP084540 (plasmid). The PacBio and Illumina raw data are accessible from the SRA under the accession numbers SRR18010266 (PacBio) and SRR18010265 (Illumina).
